# First-Principles Study on the Alloying Segregation and Ideal Fracture at Coherent B2-NiAl and BCC-Fe Interface

**DOI:** 10.3390/ma18081805

**Published:** 2025-04-15

**Authors:** Hui Chen, Yu Wang, Jianshu Zheng, Chengzhi Zhao, Qing Li, Xin Wei, Boning Zhang

**Affiliations:** 1 AECC Beijing Institute of Aeronautical Materials, Beijing 100095, China; 13810586986@163.com; 2 School of Materials Science and Engineering, University of Science and Technology Beijing, Beijing 100083, China; zhjsh2010@163.com; 3 Zhonghang Shangda Superalloys Co., Ltd., Xingtai 054800, China; zhaochengzhi1@163.com (C.Z.); liqing6744@163.com (Q.L.); 18233225652@163.com (X.W.)

**Keywords:** interface, B2-NiAl, high strength steel, first-principles calculation, alloy design

## Abstract

Nano-precipitates play a vital role in the development of ultra-high strength steels (UHSSs). In recent decades, the B2-NiAl phase, which forms highly coherent interfaces with the BCC-Fe matrix, has attracted significant attention for enhancing the strength of UHSSs. However, direct experimental investigation of alloying elements—specifically their atomic distribution and the resulting effects on the interfacial bonding strength of nano-precipitates—remains challenging. This study uses density functional theory (DFT)-based first-principles calculations to investigate the role of alloying elements in modifying interfacial characteristics. Six elements—Al, Ni, Co, Cr, Mo, and C—are introduced at various occupation sites within the coherent interface model to calculate the formation energy. The predicted preferential distribution of solid-solution atoms aligns well with experimental findings. Stable configurations of alloying segregation are selected for first-principles rigid tensile fracture tests along the <001> direction. Electronic structure analysis reveals that Co, Cr, and Mo segregation enhances interface strength due to solute-induced high charge density and the preservation of bonding characteristics of bulk phases at the interface. The results offer valuable insights and practical guidance for developing novel ultrahigh-strength structural steels strengthened by B2-NiAl.

## 1. Introduction

Ultra-high strength steels (UHSSs) are crucial structural materials in modern industries. Achieving optimal mechanical properties in high-performance UHSSs depends heavily on nano-scale precipitates, which can generally be classified into carbides/carbon-nitrides (e.g., the NaCl-type MC carbides composed of microalloying elements such as Nb, Ti, and V) and intermetallic compounds (e.g., the B2-NiAl, L_12_-Ni_3_Al, and Cu-rich phases) [1]. Compared to other microstructural features including grain boundaries and matrix-dissolved solute atoms, coherent nano-precipitates are widely recognized for their substantial contribution to strength enhancement. A prominent example is the B2-NiAl phase, featuring high interfacial coherency, high number density, and dislocation-cutting mechanism to enable strength up to 2.2 GPa in managing UHSSs [2,3]. However, while these precipitates increase strength, they can significantly reduce the ductility of precipitation-hardened UHSSs [4]. Additionally, under rapid impact loading, the numerous B2-NiAl nano-precipitates within the BCC-Fe matrix can promote cleavage fractures and drastically reduce toughness [5].

Extensive studies have investigated the trade-off between strength and ductility/toughness in B2-NiAl-strengthened ultra-high strength steels (UHSSs) [6,7,8]. The mechanism behind the relatively low ductility in the steels has been attributed to the precipitation of B2-NiAl, which depletes Ni from the matrix and promotes Cu segregation at the grain boundaries, both of which contribute to embrittlement [9]. Additionally, the high strain incompatibility between the B2-NiAl nano-precipitates and the surrounding matrix leads to localized stress concentrations at their interfaces, which can result in brittle fracture. Zhang et al. reported that due to the low shear modulus and fracture energy of B2-NiAl, the Fe/NiAl coherent interfaces are prone to crack initiation, exhibiting minimal crack propagation energy or plastic deformation [5]. These findings highlight the importance of controlling interfacial characteristics to improve resistance to crack formation at coherent interfaces.

In recent decades, engineering alloying segregation at grain boundaries and phase boundaries to improve mechanical properties has attracted significant attention [10,11,12]. The thermodynamically favorable segregation of certain solute atoms at two-dimensional defects can refine grains, increase the precipitation density, and enhance the local bonding strength. For instance, Mo and W have been used to strengthen grain boundaries in UHSSs [13], and Ta and Re have been applied to strengthen the interfaces between Ni_3_Al and the FCC matrix in superalloys [14]. A systematic first-principles study has shown that common alloying elements tend to segregate at the BCC-Fe/B2-NiAl interfaces, influencing the nucleation parameters [15]. Previous experimental studies have demonstrated that Mo segregates at the Fe/NiAl interfaces [2] and suppresses the coarsening process [16]. A similar precipitate-refining role was observed for Cr segregation at the interfaces [17]. However, although first-principles calculations suggest that Mn partitioning into B2-NiAl dramatically decreases interfacial cohesion [5], the effects of other common elements, such as Mo and Cr, on interfacial strength are not yet fully understood.

In this study, density functional theory (DFT) calculations were employed to investigate the segregation behavior of typical alloying elements and their individual effect on the fracture strength of BCC-Fe/B2-NiAl coherent interface. First, the segregation energies of alloying solutes (Co, Cr, Mo, and C) and NiAl-forming elements (Ni and Al) at various sites were calculated. Second, the ideal fracture energies and tensile properties were evaluated using ab initio rigid tension tests. Finally, the mechanisms governing ideal fracture behavior were analyzed by examining differential charge density and the electron localization function (ELF). The results provide essential data, which are currently unavailable from direct experiments, to support the simulation of B2-NiAl precipitation and the enhancement of mechanical properties in B2-NiAl precipitation-hardened UHSSs.

## 2. Methodology

### 2.1. Details of DFT Calculations

The DFT-based first-principles calculations were performed on the Vienna Ab initio Simulation Package (VASP 5.4.4) [18] with the projector augmented wave (PAW) method [19,20] and the Perdew–Bruke–Ernzerhof (PBE) generalized approximation (GGA) [21,22]. The GGA-PBE gradient corrected functional reliably predicts the energetic and structural properties, aligning well with previous calculations and experimental studies [15,23,24]. The cut-off energy of 500 eV, the Monkhorst–Pack k-point meshing [25] with a density of 0.02 Å^−1^, the Methfessel–Paxton smearing [26] with a width of 0.1 eV, and spin polarization were used. The atomic relaxation and self-consistent criterion were 0.01 eV⋅Å^−1^ and 10^−6^ eV, respectively.

The elastic constants of the relaxed bulk supercells were calculated based on the energy-strain approach [27]. They are determined by the second-order derivatives of the total energy with respect to strain:

(1) $$C_{ijkl}=\frac{1}{\Omega }{\left(\frac{\partial^{2}E(\epsilon )}{\partial \epsilon_{ij}\epsilon_{kl}}\right)}_{\epsilon =0},$$

where Ω is the volume of the structure, E(ϵ) is the total energy of the structure, and $\epsilon_{ij}$ and $\epsilon_{kl}$ are the strain tensor. Compared with other methods such as the force-strain approach, the energy-strain approach was chosen due to its less strict requirement on k-point mesh density and cut-off energy. Then the bulk modulus (B), shear modulus (G), and Young’s modulus (E) were calculated according to the Voigt-Reuss-Hill (VRH) approximation [28].

### 2.2. The BCC-Fe/B2-NiAl Interface Model

**Figure 1:**
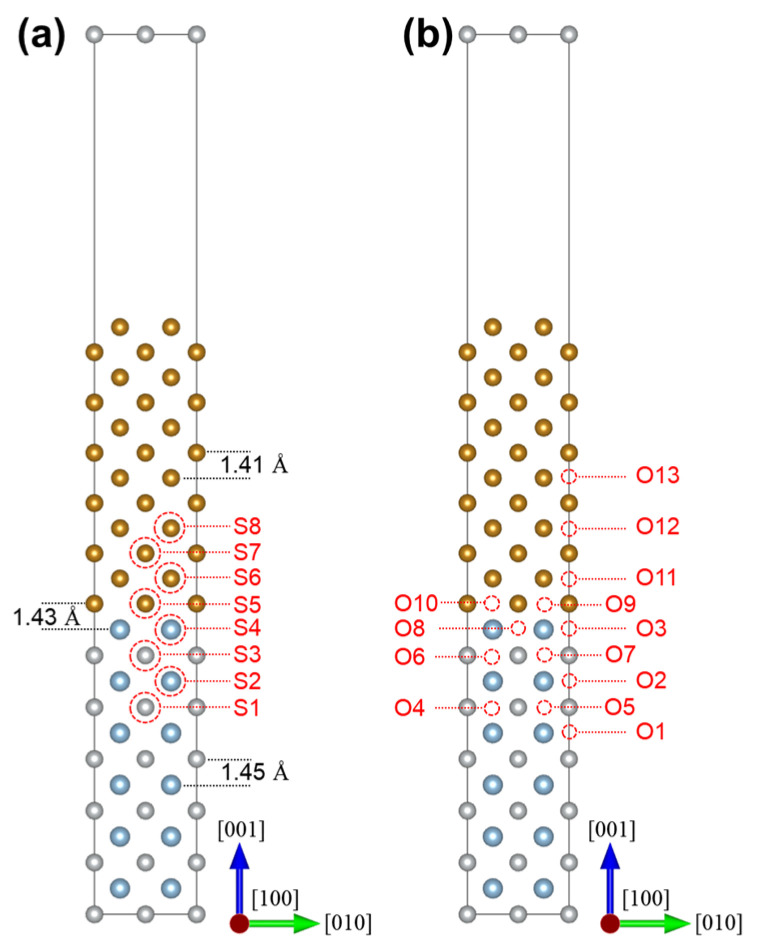
The atomic structures for the BCC-Fe/B2-NiAl coherent interface. (**a**) The possible substitutional sites (S1~S8) for Ni, Al, Co, Cr, and Mo. (**b**) The possible interstitial sites (O1~O13) for C. Only Fe (yellow spheres), Al (cyan spheres), and Ni (grey spheres) atoms are shown for simplicity.

Figure 1 shows the (001)-oriented coherent interface between BCC-Fe and B2-NiAl. Each phase consists of 12 slabs of the (001) plane. The initial models were constructed by placing the BCC-Fe (cubic, Im-3m, 229) supercell upon B2-NiAl (cubic, Pm-3m, 221). The common lattice parameters, i.e., the length parallel to the interface, were set to the average of the optimized lattice parameters of the two phases. Then the initial interlayer distance, as shown in Figure 1a, was set according to the biaxial strain and the passion ratio. A vacuum layer of 15 Å thickness was added along the [001] direction to prevent interactions between the interfaces and their periodic images. At the interface, Al atoms (blue spheres) are coordinated with four Fe atoms (yellow spheres) as their first nearest neighbors. This Al-terminated interface configuration was found to result in the lowest interfacial energy, thereby stabilizing the interface model compared to other configurations [5]. Additionally, the model meets the mechanical stability criterion. Therefore, it was selected to investigate the role of solute segregation in influencing the ideal fracture energies.

Figure 1a illustrates the possible substitutional sites for Cr, Co, and Mo, labeled as S1~S8. Within the NiAl phase, substitutional sites can either be Al sites (S2 and S4), or Ni-sites (S1 and S3). Anti-site defects, denoted as ${Ni}_{Al}$ and ${Al}_{Ni}$ using Kröger–Vink notation, are also considered. Figure 1b presents the possible interstitial sites for C, labeled O1~O13. These are octahedral sites with various metallic atoms as their nearest neighbors. Notably, the interfacial octahedral site is coordinated by different atomic species. For instance, the short axis of the flattened octahedral interstitial O9 is composed of Al and Fe, oriented along the [001] crystallographic direction, while that of O10 consists of two interfacial Fe atoms, aligned along the [010] direction. The distinct coordination environments also exist for the O4/O5, O6/O7, and O8/O3 pairs, wherein the O4, O6, and O8 are located at 1/4 of the supercell along [100] direction, while O5, O7 and O3 are situated at 1/2 of the supercell along the same [100] direction.

After fully relaxing the supercell’s volume, shape, and ionic positions, we evaluated the site occupancy preference in both the bulk B2-NiAl (4 × 4 × 4) supercell and the interface supercell by calculating the system’s formation energy ∆U, defined as

(2) $$∆U=\frac{E_{sys}-N_{Fe}\times \mu_{Fe}-N_{Ni}\times \mu_{Ni}-N_{Al}\times \mu_{Al}-x\mu_{Sol}}{N_{Fe}+N_{Ni}+N_{Al}+x},$$

where $E_{sys}$ is the total energy of the supercell with or without a solute atom, $\mu_{Fe}$, $\mu_{Ni}$, $\mu_{Al}$, and $\mu_{Sol}$ are the atomic energies of elements in their most stable crystal structures, and $N_{Fe}$, $N_{Ni}$, $N_{Al}$, and x are the corresponding number of atoms in the interface supercell.

### 2.3. Ideal Fracture Energies

First-principles tensile tests were performed on the relaxed supercells with solutes located at the preferred segregation sites. The (001) plane at the interface between the BCC-Fe and B2-NiAl phases was selected as the cleavage plane. By separating the crystals and performing self-consistent calculations, the system’s energy, E(x), as a function of the separation distance, x, was obtained. The first-principles data were then fitted using the Rose equation [29]:

(3) $$\frac{E\left(x\right)-E\left(0\right)}{A}=W_{sep}-W_{sep}(1+\frac{x}{\lambda })e^{\left(-x/\lambda \right)},$$

where $E\left(0\right)$ is the energy of the interface model without rigid separation, A is the area of the interface, $W_{sep}$ is the ideal fracture energy (work of separation), and λ is the critical separation distance.

## 3. Results and Discussions

### 3.1. Solute Occupancy in B2-NiAl Bulk and Its Effect on Elastic Properties

**Figure 2:**
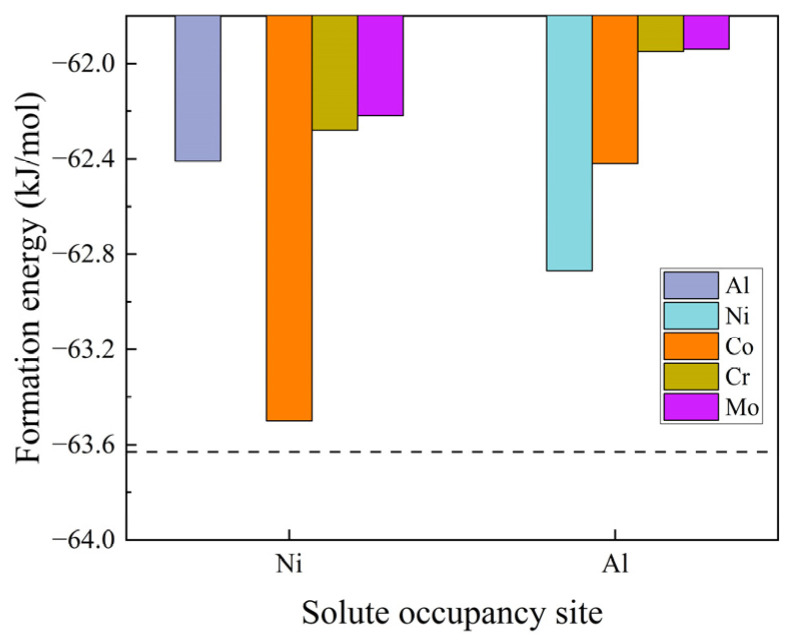
The formation energies for substitutional solutes occupying the Ni and Al sites in B2-NiAl bulk. The dashed line indicates the case for B2-NiAl without alloying solutes.

After performing full structural relaxations, the preferred occupation sites for alloying atoms in the B2-NiAl bulk were calculated. Figure 2 compares the system formation energies (∆U) for substitutional alloying solutes occupying the Ni and Al sites in the B2-NiAl bulk. Introducing a single solute atom into the (4 × 4 × 4) supercell increases the formation energy, suggesting that the precipitates tend to expel these solute atoms in order to achieve the equilibrium composition with the highest stability. Additionally, the anti-site defects of Al and Ni lead to higher formation energies, which destabilize the structure. Among the typical alloying elements in UHSSs, Co, Cr, and Mo are energetically more favorable to occupy the Ni site than the Al site within B2-NiAl.

The formation energies of a C atom in two octahedral interstitial sites were calculated. The results show that C is more likely to occupy the octahedral site surrounded by four second-nearest-neighbor (NN) Ni atoms ($C_{i,1}$, −61.35 kJ/mol) than the site with four 2NN Al atoms ($C_{i,2}$, −61.25 kJ/mol). Although the energy difference is small, C increases the formation energy, similar to the behavior of substitutional atoms. Therefore, the solid-solution capacity of C atoms in the B2-NiAl bulk is very low [28]. During the growth of B2-NiAl precipitates, C atoms are expected to be repelled to the BCC-Fe matrix.

For BCC-Fe and B2-NiAl bulk, there are three independent elastic constants ($C_{11}$, $C_{12}$ and $C_{44}$). Table 1 lists the elastic constants for both phases, calculated using the strain-energy approach [27]. The results indicate that BCC-Fe is stiffer than B2-NiAl under elastic deformation. Due to its larger lattice parameter, B2-NiAl undergoes lattice compression when forming a coherent interface with BCC-Fe. The elastic constants of B2-NiAl are $C_{11}\;$= 199.13 GPa, $C_{12}\;$= 143.79 GPa, and $C_{44}\;$= 119.11 GPa. Therefore, the stiffness of B2-NiAl is higher in the <100> direction than in shear deformation. Furthermore, the empirical criterion based on Cauchy pressure $C_{12}-C_{44}>0$ suggests the structure is dominated by metallic bonding and ductile [30]. These findings are consistent with previous data [31].

The shear modulus G is correlated with the fracture strength $\sigma_{c}$ according to the cleavage criterion of metallic materials expressed as

(4) $$\sigma_{c}=\frac{2G\gamma_{s}}{k_{y}\sqrt{d}},$$

where $\gamma_{s}$ is the surface energy, $k_{y}$ is the Hall-Petch factor, and d is the grain diameter. Based on this equation, previous studies suggest that the low shear modulus of B2-NiAl and B2-Ni(Al,Mn) contributes to the intrinsic low ductility of NiAl-strengthened steels [5]. Thus, we further investigated the elastic properties of alloyed B2-NiAl.

Table 1 shows that when anti-site defects or substitutional solutes are added at dilute concentrations to the most stable sites of B2-NiAl, the cubic symmetry is preserved, with the principal diagonal element $C_{11}=C_{22}=C_{33}$, the shear diagonal elements $C_{44}=C_{55}=C_{66}$, and the shear non-diagonal elements $C_{12}=C_{13}=C_{23}$. In contrast, when a C atom occupies an octahedral interstitial site, the structure adopts a tetragonal symmetry, resulting in two additional independent elastic constants, namely $C_{33}\neq C_{11}$ and $C_{66}\neq C_{44}$. Using these elastic constants, the bulk modulus (B), shear modulus (G), and Young’s modulus (E) were calculated. We observe that solute additions have only a minor effect on the bulk modulus. However, solutes generally decrease the shear modulus and Young’s modulus, except for ${Co}_{Ni}$ and $C_{i,1}$. Notably, as Co only slightly increases the system formation energy, its dissolution and the associated increase in shear modulus may positively influence the fracture properties of B2-NiAl, based on the cleavage fracture criterion for metallic materials [32].

### 3.2. Solute Segregation at the Coherent Interface

**Figure 3:**
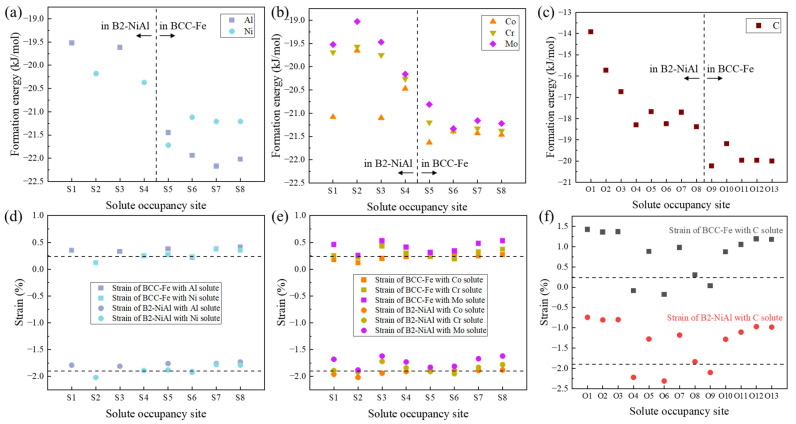
Calculation results of solute segregation and the lateral strains in the two phases. The formation energies of interface systems with the excess (**a**) Al and Ni, (**b**) Co, Cr, and Mo, and (**c**) C atom. The effect of solute (**d**) Al and Ni, (**e**) Co, Cr, and Mo, (**f**) C atom on the lateral strain of Fe phase (squares) and NiAl phase (circles). The dashed lines indicated the case without solute atom.

To assess the tendency for solute segregation, the formation energies of solutes occupying various sites in the interface supercell, calculated using Equation (2), are shown in Figure 3. When B2-NiAl precipitates deviate from ideal stoichiometry, excess Al atoms (Al anti-site defects) tend to accumulate near the coherent interface (Al_S3). In contrast, excess Al atoms in the Fe matrix tend to move away from the interface, thereby reducing the system formation energy. Excess Ni atoms in the B2-NiAl phase also prefer to be located near the interface (Ni_S4). In the Fe matrix, excess Ni atoms are more likely to accumulate at the interface, forming a Ni-terminated interface. This promotes the growth of the B2-NiAl precipitate phase.

Figure 3b shows that substitutional Co, Cr, and Mo atoms occupying the Ni sublattice of NiAl (S1) result in lower energies compared to occupying Al positions (S2). This is consistent with the alloying dissolution behavior observed in B2-NiAl bulk, as shown in Figure 2. Due to the chemical and structural effects of the coherent interface, Cr and Mo segregate to the interfacial Al site (S4), significantly reducing the energy. In the BCC-Fe matrix, Co tends to occupy the interface layer (S5), while Cr and Mo prefer the second layer near the interface (S6). For interstitial C, Figure 3c shows that it is more favorable for C to segregate in the BCC-Fe matrix than in B2-NiAl, with C specifically tending to segregate at the interface (O9).

Experimental observations have shown that Mo [2] and Cr [17] segregate at the Fe/NiAl interfaces. Additionally, the interfaces of B2-NiAl precipitates can assist in the heterogeneous nucleation of M_2_C carbides, suggesting that C may enrich near the NiAl particles [33]. In this context, the gradual enrichment of C at the interface can serve as the precursor of carbide nucleation. The electronic state may facilitate the segregation of more C atoms until reaching some specific segregation pattern, then structural transformation occurs from the continuous cubic lattice to local HCP structures extending at least several atomic planes. The previous experimental findings are supported by current first-principles calculations, which reveal that the formation energies decrease as solutes approach the interface, with systems containing interface-segregated solutes exhibiting the lowest energy.

As a result of solute segregation, the lattice misfit at the interface is altered. Figure 3d–f summarize the average biaxial strains of the two phases for the solute-added interface models. It is observed that the Fe lattice undergoes biaxial tensile strain (upper dashed line), while the NiAl lattice experiences biaxial compressive strain (lower dashed line). These differences arise from the distinct moduli and lattice parameters of the two phases. Adding solutes slightly affects the strain when the solute is a substitutional alloying atom, whereas excess interstitial C atoms significantly alter the strain. Except for a few cases where the lattice parameters decrease, the solutes generally cause lattice expansion, as indicated by the data points above the dashed lines. Notably, at relatively stable segregation sites, solutes tend to maintain or only slightly modify the original strain state. Since compressive strains generally increase energy more than tensile strains [34], the slight changes in strain compatibility suggest that the chemical interactions between interfacial atoms also play a role in stabilizing the interface.

### 3.3. The Effect of Solute Segregation on Ideal Fracture at Interface

**Figure 4:**
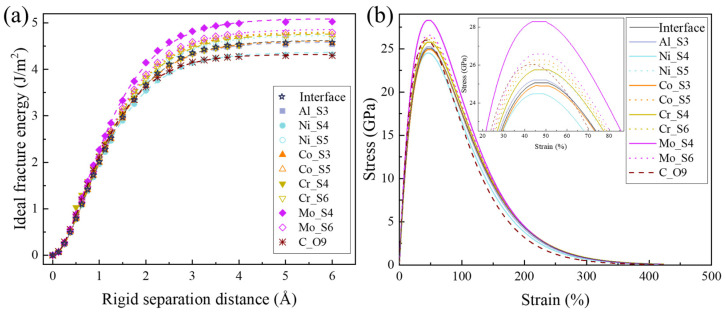
First-principles tensile test results. (**a**) The ideal fracture energy as a function of the rigid separation distance and fitting to the Rose equation. (**b**) The stress–strain curves.

The ideal fracture energy (work of separation) during the rigid tensile process was simulated using the stable interface model of BCC-Fe and B2-NiAl. Through the above calculations of formation energies at various atomic sites, the two most favorable occupation configurations for interface segregation of Ni, Co, Cr, and Mo, exhibiting the lowest formation energies, were used to calculate the ideal fracture strength. First-principles data, shown in Figure 4a with hollow stars for the interface and other symbols for the solute-segregated interfaces, were satisfactorily fitted using the Rose equation [29]. The fitting curves display a rapid increase in energy at the initial stage, followed by a slower increase until the steady-state energy is reached. Figure 4b shows the first derivative of the rigid tensile curve, which represents the stress–strain relationship. The ideal work of separation ($W_{sep}$) for the BCC-Fe/B2-NiAl interface is 4.63 J/m^2^, the theoretical tensile strength ($\sigma_{max}$) is 25.09 GPa, and the critical strain ($\varepsilon_{cri}$) is 47.94%. Compared to the BCC-Fe matrix ($W_{sep}\;$= 5.07 J/m^2^, $\sigma_{max}\;$= 32.77 GPa) and the B2-NiAl precipitate phase ($W_{sep}\;$= 4.87 J/m^2^, $\sigma_{max}\;$= 26.34 GPa), the coherent interface shows a higher tendency for theoretical fracture.

Table 2 compares the ideal fracture parameters at the interface with and without solute segregation. It is found that the presence of Al anti-site defect (Al_S3) or Ni anti-site defect (Ni_S4) near the interface tends to decrease the work of separation, tensile strength, and critical strain. In contrast, when Ni atoms in the BCC-Fe matrix segregate at the interface (Ni_S5), the theoretical tensile fracture strength increases. This configuration can be considered a coherent interface structure with Ni as the terminal atom. However, first-principles rigid tensile tests indicate that the ideal fracture occurs more readily at the intermediate plane between the segregated Ni and BCC-Fe. Substitutional Co occupying the Ni sublattice (Co_S3) in B2-NiAl at the interface has a minimal effect on interface strength. When substitutional Co, Cr, and Mo atoms occupy more stable, low-energy sites at the interface (Co_S5, Cr_S4, Cr_S6, Mo_S4, and Mo_S6), they can enhance the theoretical tensile strength, with Mo showing the most pronounced effect. For interstitial C, the most stable segregation site at O_9 reduces the energy required for rigid tensile fracture. The DFT results strongly support previous experimental findings regarding the enhancement of mechanical properties through alloying additions [2,16,17].

### 3.4. The Electronic Structures at the Interface

**Figure 5:**
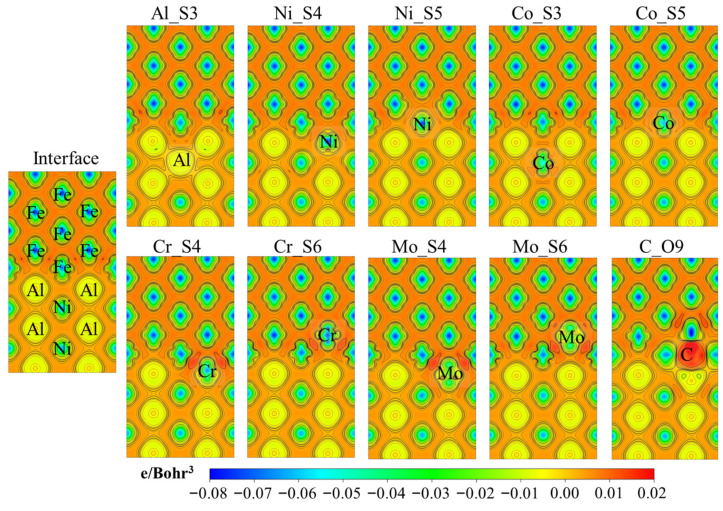
The differential charge density of the various interface systems projected on the (110) plane.

The differential charge density reveals information about charge redistribution before and after atomic bonding formation. Figure 5 presents the differential charge density of the BCC-Fe/B2-NiAl interface, projected onto the (110) plane. The irregular blue-green regions in the upper half of the figure correspond to BCC-Fe atoms near the interface, while the irregular blue-green regions and near-circular yellow regions in the lower half correspond to Ni and Al atoms, respectively. A notable feature of the interface region is the charge accumulation between the interfacial Fe and Al atoms, which stabilizes the interface with low energy. Additionally, charge accumulation is observed between Fe atoms in the first and second interface layers of the BCC-Fe. This may weaken atomic bonding strength in the interface region, thereby increasing the fracture sensitivity of the interface compared to the bulk phases of BCC-Fe and B2-NiAl.

Figure 5 shows the differential charge density for interface systems with solute atoms. The local regions corresponding to the atoms, aside from the labeled solute atoms, are as described above. Regions between the Al anti-site defect (Al_S3) and its neighboring Al atoms exhibit electron loss. The color in the charge accumulation zone between Fe atoms at the interface and those in the second interface layer lightens, indicating a reduced charge accumulation. For the interface segregation of Ni (Ni_S4 and Ni_S5), a decrease in charge accumulation is observed between the interfacial Fe and Al atoms. Similarly, charge accumulation between Fe atoms at the interface and in the second interface layer also decreases.

When Co substitutes Al in the second interface layer of B2-NiAl (Co_S3), the primary effect is on the local charge redistribution between Al atoms near the interface. For substitutional solutes such as Co, Cr, and Mo at sites favorable for enhancing theoretical fracture strength (Co_S5, Cr_S4 and Cr_S6, Mo_S4 and Mo_S6), the differential charge densities show a common trend: the solute atoms increase the electronic density in the interface region. This suggests that the introduction of solute atoms not only stabilizes the interface but also may enhance its mechanical properties. Among these, Mo has the most pronounced effect, likely due to its higher ability to donate electrons and increase the electronic density at the interface. For interstitial C at the O9 site, the nearby metallic atoms lose more electrons due to covalent bonding, leading to significant charge distribution inhomogeneity in the interface region. Additionally, red-colored regions representing charge accumulation are observed between the neighboring and second-neighboring atoms of C. This phenomenon indicates that charge interactions are stronger than in the cases of substitutional solutes.

**Figure 6:**
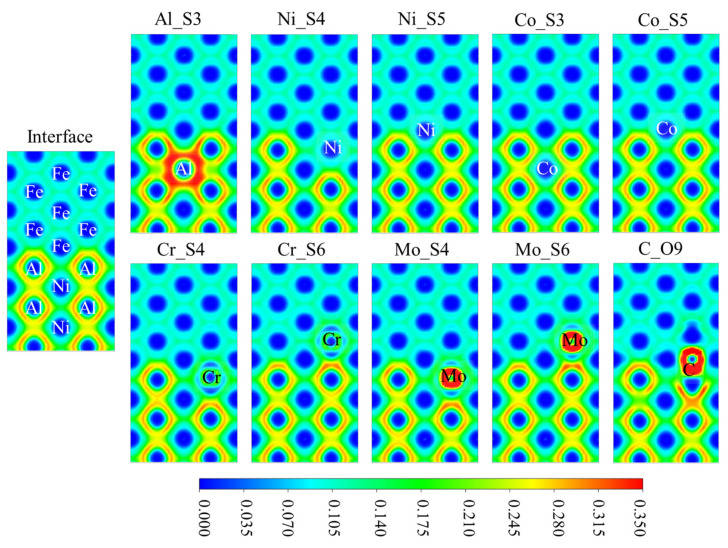
The electron localization function (ELF) of the various interface systems projected onto the (110) plane.

To directly observe the bonding characteristics at the interfaces, the ELF results are presented in Figure 6. Projected onto the (110) plane, the upper circular dark-blue regions correspond to BCC-Fe atoms, while the lower circular dark-blue regions correspond to Ni atoms. The lower circular dark-blue region, enclosed by orange-yellow high ELF regions, corresponds to Al atoms. In the alloyed system with the Al_S3 defect, wide orange-red regions with a high ELF are observed near the anti-site defect. This system shows a notable reduction in ideal fracture energy during rigid tensile testing, suggesting that the bonding significantly embrittles the interface. Substituting Al at the interface with Ni, Cr, or Mo at the S4 site eliminates the “ring-shaped” high ELF regions. The resulting ELF distribution is similar to that observed in the interior of BCC-Fe, which may help maintain the bulk phase properties. In contrast, when substitutional solute atoms accumulate on the BCC-Fe side, the “ring-shaped” high ELF feature of the B2-NiAl bulk phase is retained, particularly at the Cr_S6 and Mo_S6 interfaces, where electronic localization is more pronounced at the center of the two-phase interface. For interstitial C occupying the octahedral O7 site, the local ELF is high, disrupting the continuity of the high ELF region within the B2-NiAl phase at the interface.

The preferential distribution of solute atoms and their impact on the theoretical fracture behavior during tensile deformation is directly influenced by the local electronic structures at the interface [35]. Based on the above analysis, we conclude that when the interaction between solute atoms and their neighboring atoms increases the charge density and maintains the electronic structure characteristics of the bulk phases in the interface region, the theoretical fracture strength of the BCC-Fe/B2-NiAl interface during rigid tensile deformation can be enhanced.

## 4. Conclusions

In this study, first-principles calculations were performed to gain atomic-level insights into the BCC-Fe/B2-NiAl coherent interface, which is crucial for the development of precipitation-hardened UHSSs. The results indicate that Co, Cr, Mo, and C tend to segregate at the interface, and anti-site defects formed by excess Al and Ni also favor segregation toward the interface. These atomic-scale predictions align well with existing experimental observations. First-principles rigid tensile tests show that interfacial segregation of substitutional Co, Cr, or Mo increases the ideal fracture energy and theoretical fracture strength, with Mo being the most effective. The beneficial effect can be attributed to solute-induced charge density enhancement and the preservation of bonding characteristics of the two-phase bulk at the interface. These findings provide valuable guidance for developing B2-NiAl-strengthened UHSSs with improved macroscopic ductility and toughness.

## Figures and Tables

**Table 1:** The effect of alloying on the elastic properties of B2-NiAl bulk.

Bulk	$C_{11}$ (GPa)	$C_{12}$ (GPa)	$C_{33}$ (GPa)	$C_{44}$ (GPa)	$C_{66}$ (GPa)	B (GPa)	G (GPa)	E (GPa)
BCC-Fe	276.65	150.02	276.65	101.98	101.98	192.23	84.24	220.49
B2-NiAl	199.13	143.79	199.13	119.11	119.11	162.24	66.92	175.47
${Al}_{Ni}$	195.14	143.09	195.14	115.80	115.80	160.44	64.27	169.06
${Ni}_{Al}$	198.51	145.20	198.51	120.25	120.25	162.97	66.41	174.28
${Co}_{Ni}$	201.38	143.25	201.38	120.08	120.08	162.62	68.49	179.23
${Cr}_{Ni}$	183.01	153.44	183.01	119.94	119.94	163.29	54.54	144.79
${Mo}_{Ni}$	193.99	145.95	193.99	117.31	117.31	161.96	62.97	165.98
$C_{i,1}$	201.34	142.63	139.76	206.13	111.61	161.45	67.25	176.51

**Table 2:** The ideal work of separation ($W_{sep}$), critical separation distance (λ), theoretical tensile strength ($\sigma_{max}$), and critical strain ($\varepsilon_{cri}$) of the interface systems without/with solute segregation.

System	$W_{sep}$ (J/m^2^)	λ (Å)	$\sigma_{max}$ (GPa)	$\varepsilon_{cri}$
Interface	4.63	0.678	25.09	47.94
Al_S3	4.59	0.669	25.23	47.24
Ni_S4	4.37	0.656	24.50	46.32
Ni_S5	4.73	0.675	25.76	47.72
Co_S3	4.62	0.681	24.94	48.13
Co_S5	4.81	0.672	26.30	47.51
Cr_S4	4.77	0.680	25.79	48.01
Cr_S6	4.79	0.675	26.07	47.72
Mo_S4	5.09	0.661	28.33	46.72
Mo_S6	4.86	0.672	26.61	47.49
C_O7	4.33	0.611	26.02	43.20

## Data Availability

The raw data supporting the conclusions of this article will be made available by the authors on request.

## References

[1] Sankaran K.K., Mishra R.S., Sankaran K.K., Mishra R.S. (2017). Chapter 6—Ultrahigh Strength Steels. Metallurgy and Design of Alloys with Hierarchical Microstructures.

[2] Jiang S., Wang H., Wu Y., Liu X., Chen H., Yao M., Gault B., Ponge D., Raabe D., Hirata A. (2017). Ultrastrong steel via minimal lattice misfit and high-density nanoprecipitation. Nature.

[3] Wang Z.H., Niu B., Wang Q., Dong C., Jie J.C., Wang T.M., Nieh T.G. (2021). Designing ultrastrong maraging stainless steels with improved uniform plastic strain via controlled precipitation of coherent nanoparticles. J. Mater. Sci. Technol..

[4] Sun L., Simm T.H., Martin T.L., McAdam S., Galvin D.R., Perkins K.M., Bagot P.A.J., Moody M.P., Ooi S.W., Hill P. (2018). A novel ultra-high strength maraging steel with balanced ductility and creep resistance achieved by nanoscale β-NiAl and Laves phase precipitates. Acta Mater..

[5] Zhang X., Wang J., Zhou T., Yan L., Yu H. (2024). Impact toughness and fracture propagation mechanism of NiAl precipitation-strengthened HSLA steels. Mater. Des..

[6] Zhou B.C., Yang T., Zhou G., Wang H., Luan J.H., Jiao Z.B. (2021). Mechanisms for suppressing discontinuous precipitation and improving mechanical properties of NiAl-strengthened steels through nanoscale Cu partitioning. Acta Mater..

[7] Yang X., Di X., Duan Q., Fu W., Ba L., Li C. (2023). Effect of precipitation evolution of NiAl and Cu nanoparticles on strengthening mechanism of low carbon ultra-high strength seamless tube steel. Mater. Sci. Eng. A.

[8] Sahoo B.K., Srivastava V.C., Chandan A.K., Ghosh Chowdhury S. (2022). Enhancing the properties of Al–Ni added medium Mn steel by tailoring B2–NiAl precipitates through aging treatment. Mater. Sci. Eng. A.

[9] Kapoor M., Isheim D., Vaynman S., Fine M.E., Chung Y.W. (2016). Effects of increased alloying element content on NiAl-type precipitate formation, loading rate sensitivity, and ductility of Cu- and NiAl-precipitation-strengthened ferritic steels. Acta Mater..

[10] Zhang B., Xiong K., Wang M., Liu Z., Shen K., Mao Y., Chen H. (2024). Grain boundary alloying segregation to resist hydrogen embrittlement in BCC-Fe steels: Atomistic insights into solute-hydrogen interactions. Scr. Mater..

[11] Mai H.L., Cui X.-Y., Scheiber D., Romaner L., Ringer S.P. (2022). The segregation of transition metals to iron grain boundaries and their effects on cohesion. Acta Mater..

[12] Wang J., Enomoto M., Shang C. (2020). First-principles study on the interfacial segregation at coherent Cu precipitate/Fe matrix interface. Scr. Mater..

[13] Geng W.T., Freeman A.J., Olson G.B. (2001). Influence of alloying additions on grain boundary cohesion of transition metals: First-principles determination and its phenomenological extension. Phys. Rev. B.

[14] Ahmed F.A.M., Xue H.-T., Tang F.-L., An J.-P., Luo Y.-Q., Lu X.-F., Ren J.-Q. (2020). Segregation of alloying elements and their effects on the thermodynamic stability and fracture strength of γ-Ni/γ′-Ni3Al interface. J. Mater. Sci..

[15] Gao X., Wang H., Ma C., Lv M., Ren H. (2021). Segregation of alloying elements at the bcc-Fe/B2–NiAl interface and the corresponding effects on the interfacial energy. Intermetallics.

[16] Calderon H.A., Fine M.E., Weertman J.R. (1988). Coarsening and morphology of β′ particles in Fe-Ni-Al-Mo ferritic alloys. Metall. Trans. A.

[17] Ping D.H., Ohnuma M., Hirakawa Y., Kadoya Y., Hono K. (2005). Microstructural evolution in 13Cr–8Ni–2.5Mo–2Al martensitic precipitation-hardened stainless steel. Mater. Sci. Eng. A.

[18] Kresse G., Furthmuller J. (1996). Efficient iterative schemes for ab initio total-energy calculations using a plane-wave basis set. Phys. Rev. B.

[19] Blöchl P.E. (1994). Projector augmented-wave method. Phys. Rev. B.

[20] Kresse G., Joubert D. (1999). From ultrasoft pseudopotentials to the projector augmented-wave method. Phys. Rev. B.

[21] Perdew J.P., Burke K., Ernzerhof M. (1996). Generalized gradient approximation made simple. Phys. Rev. Lett..

[22] Perdew J.P., Chevary J.A., Vosko S.H., Jackson K.A., Pederson M.R., Singh D.J., Fiolhais C. (1992). Atoms, molecules, solids, and surfaces: Applications of the generalized gradient approximation for exchange and correlation. Phys. Rev. B.

[23] Yu J., Lin X., Wang J., Chen J., Huang W. (2009). First-principles study of the relaxation and energy of bcc-Fe, fcc-Fe and AISI-304 stainless steel surfaces. Appl. Surf. Sci..

[24] Faraoun H., Aourag H., Esling C., Seichepine J.L., Coddet C. (2005). Elastic properties of binary NiAl, NiCr and AlCr and ternary Ni2AlCr alloys from molecular dynamic and Abinitio simulation. Comput. Mater. Sci..

[25] Monkhorst H.J., Pack J.D. (1976). Special points for Brillouin-zone integrations. Phys. Rev. B.

[26] Methfessel M., Paxton A.T. (1989). High-precision sampling for Brillouin-zone integration in metals. Phys. Rev. B Condens. Matter.

[27] Balasubramanian K., Manna S., Sankaranarayanan S.K.R.S. (2023). Elastemp—A workflow to compute the quasi-harmonic temperature dependent elastic constants of materials. Comput. Mater. Sci..

[28] Zuo L., Humbert M., Esling C. (1992). Elastic properties of polycrystals in the Voigt-Reuss-Hill approximation. J. Appl. Crystallogr..

[29] Rose J.H., Ferrante J., Smith J.R. (1981). Universal Binding Energy Curves for Metals and Bimetallic Interfaces. Phys. Rev. Lett..

[30] Thompson R.P., Clegg W.J. (2018). Predicting whether a material is ductile or brittle. Curr. Opin. Solid State Mater. Sci..

[31] Xing H., Dong A., Huang J., Zhang J., Sun B. (2018). Revisiting intrinsic brittleness and deformation behavior of B2 NiAl intermetallic compound: A first-principles study. J. Mater. Sci. Technol..

[32] Roesler J., Harders H., Baeker M. (2007). Mechanical Behaviour of Engineering Materials: Metals, Ceramics, Polymers, and Composites.

[33] Danoix F., Danoix R., Akre J., Grellier A., Delagnes D. (2011). Atom probe tomography investigation of assisted precipitation of secondary hardening carbides in a medium carbon martensitic steels. J. Microsc..

[34] Wang K., Shang S.-L., Wang Y., Liu Z.-K., Liu F. (2018). Martensitic transition in Fe via Bain path at finite temperatures: A comprehensive first-principles study. Acta Mater..

[35] Park N.Y., Choi J.H., Cha P.R., Jung W.S., Chung S.H., Lee S.C. (2013). First-principles study of the interfaces between Fe and transition metal carbides. J. Phys. Chem. C.

